# EnvR is a potent repressor of *acrAB* transcription in *Salmonella*

**DOI:** 10.1093/jac/dkac364

**Published:** 2022-10-29

**Authors:** Jessica M A Blair, Pauline Siasat, Helen E McNeil, Abigail Colclough, Vito Ricci, Amelia J Lawler, Hind Abdalaal, Michelle M C Buckner, Alison Baylay, Stephen J Busby, Laura J V Piddock

**Affiliations:** College of Medical and Dental Sciences, Institute of Microbiology and Infection, University of Birmingham, Birmingham B15 2TT, UK; College of Medical and Dental Sciences, Institute of Microbiology and Infection, University of Birmingham, Birmingham B15 2TT, UK; College of Medical and Dental Sciences, Institute of Microbiology and Infection, University of Birmingham, Birmingham B15 2TT, UK; College of Medical and Dental Sciences, Institute of Microbiology and Infection, University of Birmingham, Birmingham B15 2TT, UK; College of Medical and Dental Sciences, Institute of Microbiology and Infection, University of Birmingham, Birmingham B15 2TT, UK; College of Medical and Dental Sciences, Institute of Microbiology and Infection, University of Birmingham, Birmingham B15 2TT, UK; College of Medical and Dental Sciences, Institute of Microbiology and Infection, University of Birmingham, Birmingham B15 2TT, UK; College of Medical and Dental Sciences, Institute of Microbiology and Infection, University of Birmingham, Birmingham B15 2TT, UK; College of Medical and Dental Sciences, Institute of Microbiology and Infection, University of Birmingham, Birmingham B15 2TT, UK; College of Medical and Dental Sciences, Institute of Microbiology and Infection, University of Birmingham, Birmingham B15 2TT, UK

## Abstract

**Background:**

Resistance nodulation division (RND) family efflux pumps, including the major pump AcrAB-TolC, are important mediators of intrinsic and evolved antibiotic resistance. Expression of these pumps is carefully controlled by a network of regulators that respond to different environmental cues. EnvR is a TetR family transcriptional regulator encoded upstream of the RND efflux pump *acrEF*.

**Methods:**

Binding of EnvR protein upstream of *acrAB* was determined by electrophoretic mobility shift assays and the phenotypic consequence of *envR* overexpression on antimicrobial susceptibility, biofilm motility and invasion of eukaryotic cells *in vitro* was measured. Additionally, the global transcriptome of clinical *Salmonella* isolates overexpressing envR was determined by RNA-Seq.

**Results:**

EnvR bound to the promoter region upstream of the genes coding for the major efflux pump AcrAB in *Salmonella*, inhibiting transcription and preventing production of AcrAB protein. The phenotype conferred by overexpression of *envR* mimicked deletion of *acrB* as it conferred multidrug susceptibility, decreased motility and decreased invasion into intestinal cells *in vitro*. Importantly, we demonstrate the clinical relevance of this regulatory mechanism because RNA-Seq revealed that a drug-susceptible clinical isolate of *Salmonella* had low *acrB* expression even though expression of its major regulator RamA was very high; this was caused by very high EnvR expression.

**Conclusions:**

In summary, we show that EnvR is a potent repressor of *acrAB* transcription in *Salmonella*, and can override binding by RamA so preventing MDR to clinically useful drugs. Finding novel tools to increase EnvR expression may form the basis of a new way to prevent or treat MDR infections.

## Introduction

Efflux pumps export diverse antimicrobial compounds out of bacterial cells and, therefore, are an important mechanism of MDR. In *Salmonella enterica* and *Escherichia coli* AcrAB-TolC is the major efflux pump system and increased expression of this pump confers clinically relevant levels of antibiotic resistance.^1^

The regulation of *acrB* in *Salmonella* and *E. coli* is complex and involves regulation at local and global levels.^2^ The *acrB* gene is encoded alongside, but divergently from, its local transcriptional repressor *acrR*, which belongs to the TetR family.^3,4^ More recently it has been shown that the RNA binding protein CsrA is involved in the regulation of AcrAB in *S. enterica.*^5^ CsrA binds directly to the 5′ end of the *acrAB* transcript, allowing for efficient translation of the AcrAB proteins. Expression of *acrB* is also controlled by global transcription factors of the AraC-XylS family. In *E. coli* these include MarA, SoxS and Rob, while in *Salmonella* the major activator of *acrAB* expression is an additional AraC-XylS family transcription factor, RamA.^6,7^ Increased expression of these AraC-XylS transcription factors increases transcription of *acrAB*, which leads to MDR.^8,9^ These global transcription factors are under the control of locally encoded repressors of the MarR family. For example, transcription of *ramA* is repressed by binding of the local repressor RamR to the *ramA* promoter region. This inhibits transcription of the *ramA* gene, preventing induction of *acrAB* transcription above basal levels. In MDR clinical isolates of *Salmonella*, mutations have been detected in *ramR*, which prevent binding of the RamR protein to the *ramA* promoter region. This relieves transcriptional repression of *ramA* and causes increased RamA production, which activates increased *acrAB* expression and MDR.^10–13^ In addition to control at the level of transcription, the amount of RamA protein is also controlled post-translationally by the Lon protease.^14^

In *E. coli*, an additional repressor of *acrAB* expression, EnvR (or AcrS), has been described.^15^ The *envR* gene is encoded alongside and divergently from the *acrEF* operon but appears to be predominantly a repressor of *acrAB* transcription, not *acrEF*. EnvR binds to the promoter region upstream of *acrAB* and has a greater binding affinity than the local repressor AcrR and therefore has a greater effect on *acrAB* expression level.^15^ EnvR is proposed to act as a switch between *acrAB* and *acrEF* transcription; transcription is initiated concurrently with *acrEF* and represses transcription of *acrAB.*^15^

L3 and L18 are two previously described clinical isolates of *S. enterica* serovar Typhimurium collected from a single patient with an atypical *Salmonella* infection before and after a prolonged course of antimicrobial treatment.^16–18^ The pre-therapy isolate, L3, was susceptible to antibiotics and expression of *acrB* is low. Over the course of treatment, drug resistance developed and strains isolated at later stages of treatment were MDR. The mechanism of MDR in these isolates is multifactorial but includes overexpression of AcrB and a substitution in AcrB that altered its substrate specificity and made it better able to export the drugs with which the patient was being treated.^18^ We have previously reported that both L3 (pre-ciprofloxacin therapy) and L18 (isolated after 4.5 months of antibiotic treatment) have a mutation in the regulator gene *ramR*, leading to high expression of *ramA* to similar levels in both L3 and L18.^19^ However, despite high levels of *ramA* only, L18 overexpresses *acrB*/AcrB.^20^ This study aimed to further investigate the mechanism of antibiotic resistance in these clinical isolates of *Salmonella* and elucidate the molecular basis of the contrasting expression levels of AcrB.

## Materials and methods

### Bacterial strains

The origin of the clinical isolates, L3 and L18, has been previously described.^16,18^*S.* Typhimurium SL1344 and isogenic mutants were used as comparators (all strain information is in Table S1, available as Supplementary data at *JAC* Online). Genes were inactivated by homologous recombination followed by removal of the inserted resistance gene as described previously.^21^

### RNA extraction

*S*. Typhimurium strains were grown overnight in LB at 37°C and subcultured into MOPS minimal media and grown at 37°C with shaking at 180 rpm until mid-logarithmic phase. Three biological replicate RNA preparations were made from each strain using the Promega wizard SV RNA kit and quantified as described previously.^22^ RNA-Seq was performed by ARK genomics (Edinburgh).

### Purification of AcrR and EnvR protein

Genes coding for AcrR and EnvR were cloned into the pTrc vector and transformed into BL21 (DE3). Ten millilitres of overnight culture was used to inoculate 250 mL of pre-warmed LB broth supplemented with 50 mg/L ampicillin until an OD_600_ of 600 nm was reached. Expression of AcrR and EnvR protein was induced by incubating at 37°C for 5 h with 1 mM IPTG. Cells were pelleted by centrifugation at 4000 × **g** for 20 min. These pellets were either frozen overnight at −20°C or immediately processed for purification. AcrR and EnvR proteins were purified using the Ni-NTA fast start kit (QIAGEN) according to the manufacturer’s instructions for purification under native conditions. Size-exclusion chromatography was carried out at the University of Birmingham’s Protein Expression Facility (PEF). Proteins were concentrated using Amicon Ultra 4 mL centrifugal filters (Merck, UK) with a molecular-weight cut-off of 10 kDa according to the manufacturer’s instructions.

### Electrophoretic mobility shift assays (EMSA)

The promoter region of *acrAB* was amplified by PCR (primers listed in Table S2) and purified (QIAquick PCR purification kit, QIAGEN, UK). EMSAs were run using the EMSA kit from Life Technologies UK (E33075). DNA/protein mixtures were separated on 6% native polyacrylamide gels for 1 h at 200 V. After electrophoresis, gels were rinsed in 0.5 ×  Tris-borate-EDTA buffer before being stained using SYBR green.

### Site-directed mutagenesis

The relevant nucleotide substitutions were introduced into the *acrAB* promoter sequence on pMW82p*acrAB* using the QuikChange Lightning Multi Site-Directed Mutagenesis Kit (StrataGene), using the following mutagenic primers: 5′-ggatagaaacccataagtttctatcaatctaacgcctgtaaattcaccga-3′ and 5′-tcggtgaatttacaggcgttagattgatagaaacttatgggtttctatcc-3′. The mutagenic primers were designed using the web-based QuikChange Primer Design Program available at https://www.agilent.com/store/primerDesignProgram.jsp. Following site-directed mutagenesis putative candidates were confirmed by DNA sequencing (Functional Genomics, University of Birmingham).

### Real-time quantitative RT–PCR (qRT–PCR)

Primers for real-time qRT–PCR were designed using Beacon Designer 4.0 (PREMIER Biosoft, USA). Sample preparation and real-time RT–PCR was performed according to ‘minimum information for publication of quantitative real-time PCR experiments’ (MIQE) guidelines.^23^ cDNA was synthesized from 2 µg of total RNA using the Superscript III cDNA synthesis kit (Invitrogen). PCR efficiency validation experiments were carried out using 5 cDNA standards of decreasing concentrations (10, 1, 0.1, 0.01, 0.001 ng/µL) to determine PCR efficiency for the housekeeping gene 16S and each test gene. qRT–PCRs were set up in biological triplicate and technical duplicate in a Bio-Rad PCR tray using 1 µL of neat cDNA for test genes and 1 µL of a 1:1000 dilution cDNA for 16S in a 25 µL reaction containing 12.5 µL of iQ SYBR green supermix (Bio-Rad, UK), 1 µL of primers (500 nm) and 9.5 µL of sterile water. qRT–PCR was carried out in a CFX-96 Real-Time system (Bio-Rad, UK) using the following protocol: 95°C for 5 min followed by 40 plate-read cycles of 95°C for 30 s, 57.3°C for 30 s and 72°C for 30 s. Data analysis was performed using the ΔΔct method and normalized to expression of 16S.

### Measurement of antimicrobial susceptibility

The MIC of various antimicrobials was determined as recommended by EUCAST.^24^

### Accumulation of Hoechst 33342

The efflux activity of the mutants was assessed by determining the accumulation of the fluorescent dye Hoechst 33342 (Sigma, UK) as described previously.^25^ Data presented are the mean of three independent biological replicates ± SEM.

### Swimming motility

LB broth was supplemented with 0.3% agar (Difco^™^ Bacto agar, BD, USA). Twenty-five millilitres of media was added to round Petri dishes. Overnight cultures were diluted to an OD_600_ of 0.5 in PBS. Plates were inoculated using a straight wire, dipped in the bacterial culture, and stabbed into the middle of the agar plate and incubated at 37°C for 7 h. After incubation, the plates were imaged and distances measured using ImageJ software.^26^ The data were analysed by multiple comparisons method using Prism software version 7 (GraphPad, San Diego, CA, USA).

### Adhesion and invasion assays

The ability of the strains to adhere to, and invade, INT-407 (human embryonic intestine cells) was measured as previously described.^27^ Each assay was repeated a minimum of three times, with each experiment including four technical replicates per bacterial strain. The results were analysed using Student’s *t*-test and *P* values of  ≤ 0.05 were considered significant.

### Western blotting

Bacterial strains were grown in LB broth (Sigma) at 37°C with aeration until mid-logarithmic phase. Total cellular protein was obtained following sonication and samples were run on 12% Bis-Tris gels (Invitrogen) along with the Magic Mark^™^ XP molecular weight marker (Invitrogen). For subsequent immunoblotting, proteins were transferred to a polyvinylidine difluoride (PVDF) membrane (Amersham) by electrophoresis for 3 h at 4°C. The membrane was blocked with 5% non-fat milk solution. After overnight incubation with AcrB primary antibody, the proteins were visualized using HRP-linked anti-rabbit secondary antibody raised in donkey (GE Healthcare) and the ECL western blotting detection system (GE Healthcare).

## Results

### RNA-Seq reveals a potential mechanism of efflux regulation in clinical isolates

The MDR phenotype of L18 has previously been shown to rely on increased expression of *acrAB*. However, the regulatory mechanism that leads to this overexpression, compared with the pre-therapy isolate L3, is not fully understood. To further characterize the clinical *Salmonella* isolates L3 and L18 and to provide possible insight into the mechanism of regulation of *acrB* expression, the transcriptomes of SL1344, L3 and L18 were determined by RNA sequencing. These data confirmed that, as previously shown,^18^ expression of both the periplasmic adaptor protein *acrA* and the resistance nodulation division (RND) pump *acrB* was significantly higher in the post-therapy isolate L18, compared with the pre-therapy isolate L3 (3.52- and 20.26-fold, respectively) (Table 1). However, using the well-characterized type strain SL1344^28^ as a comparator showed that L3 has unusually low *acrB* levels while L18 has 2.21-fold more *acrB* than SL1344, explaining the apparent scale of difference when *acrB* levels in L3 and L18 are compared with each other.

**Table 1.** Selected gene expression changes from RNA-Seq

	Gene name	Fold change in L18 relative to L3	Adjusted *P* value	Fold change in L3 relative to SL1344	Adjusted *P* value	Fold change in L18 relative to SL1344	Adjusted *P* value
Efflux genes							
* *SL469	*acrA*	3.52	0.0006			1.95	0.03
* *SL468	*acrB*	20.26	1.08E−15	0.11	4.62E−10	2.21	0.01
* *SL2800	*emrB*	2.82	0.007	0.33	0.002		
* *SL3363	*acrE*	0.085	5.37E−11	67.80	6.22E−24	5.79	8.35E−05
* *SL3364	*acrF*	0.15	1.81E−07	7.40	1.64E-08		
* *SL1505	*smvA*	0.08	1.48E−11	0.49	0.06	0.04	1.27E−17
Regulators							
* *SL569	*ramA*			75.84	2.12E−27	66.65	3.93E−26
* *SL4513	*rob*						
* *SL3362	*envR*	0.05	6.24E−15	28.23	8.66E−19		

Only expression changes that were significantly changed are included in the table. Where no value is given there was no significant difference in expression between the two strains being compared.

The genes coding for known regulators of RND efflux systems in *Salmonella* (*ramA*, *marA*, *soxS* and *rob*) had altered expression but none could explain the difference in expression of *acrB* between the pre- and post-therapy isolates. RamA is the major regulator of *acrAB* transcription in *Salmonella.*^7^ Despite contrasting *acrB* levels, the pre- and post-therapy isolates both had similarly high levels of *ramA* expression compared with SL1344 (75.8- and 66.6-fold in L3 and L18, respectively) so could not account for the differential *acrB* expression (Table 1). L18 had moderately increased levels of *marA* and *soxS* compared with SL1344 (2.65- and 3.03-fold, respectively) but again the level of expression of these genes in L3 and L18 was not significantly different. In addition, *rob* had 1.86-fold higher expression in L18 than L3.

Interestingly, compared with SL1344, the pre-therapy isolate L3 had very high expression of genes coding for another RND efflux pump, *acrEF* (67.8- and 7.39-fold, for *acrE* and *acrF*, respectively) while the *acrEF* level in L18 was comparatively lower (0.09- and 0.15-fold for *acrE* and *acrF*, respectively) (Table 1). The *acrEF* genes are encoded alongside and divergently from the TetR family repressor *envR* (or *acrS*).^15^ Expression of *envR* was far higher in the pre-therapy isolate L3 than in the post-therapy isolate L18 or SL1344. Therefore, we hypothesized that EnvR is also a negative regulator of efflux in *Salmonella* and that increased expression of *envR* in the drug-susceptible clinical isolate was responsible for repressing expression of the major efflux pump *acrAB*.

### EnvR represses acrAB expression in Salmonella by binding to the acrAB promoter

**Figure 1.**
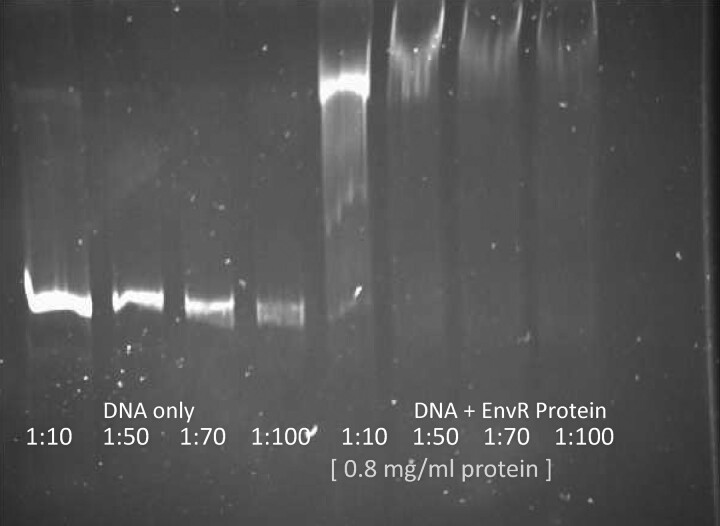
EnvR binds the promoter of *acrAB* of *Salmonella* SL1344. Purified EnvR protein (0.8 mg/mL) binds the promoter of *acrAB*, as shown by EMSA. The promoter of *acrAB* was purified and diluted to a final concentration range of 15–1.5 ng/µL. The binding of EnvR to this promoter resulted in the shift of the fluorescent band, indicating the formation of a DNA:EnvR complex. The first dilution (1:10; 15 ng/µL) was then selected as the standard DNA concentration in further EMSA experiments.

EnvR in *Salmonella* SL1344 has 69% identity with the previously studied homologue in *E. coli*. In *E. coli*, EnvR binds directly to a 30 bp region of the DNA in the promoter region of *acrAB*.^15^ Using purified EnvR protein from SL1344 and a PCR-amplified fragment containing the sequence of 200 bp upstream of *acrA* (primers in Table S2), an EMSA showed binding of *Salmonella* EnvR to the fragment causing a shift in the position of the DNA band (Figure 1).

**Figure 2.**
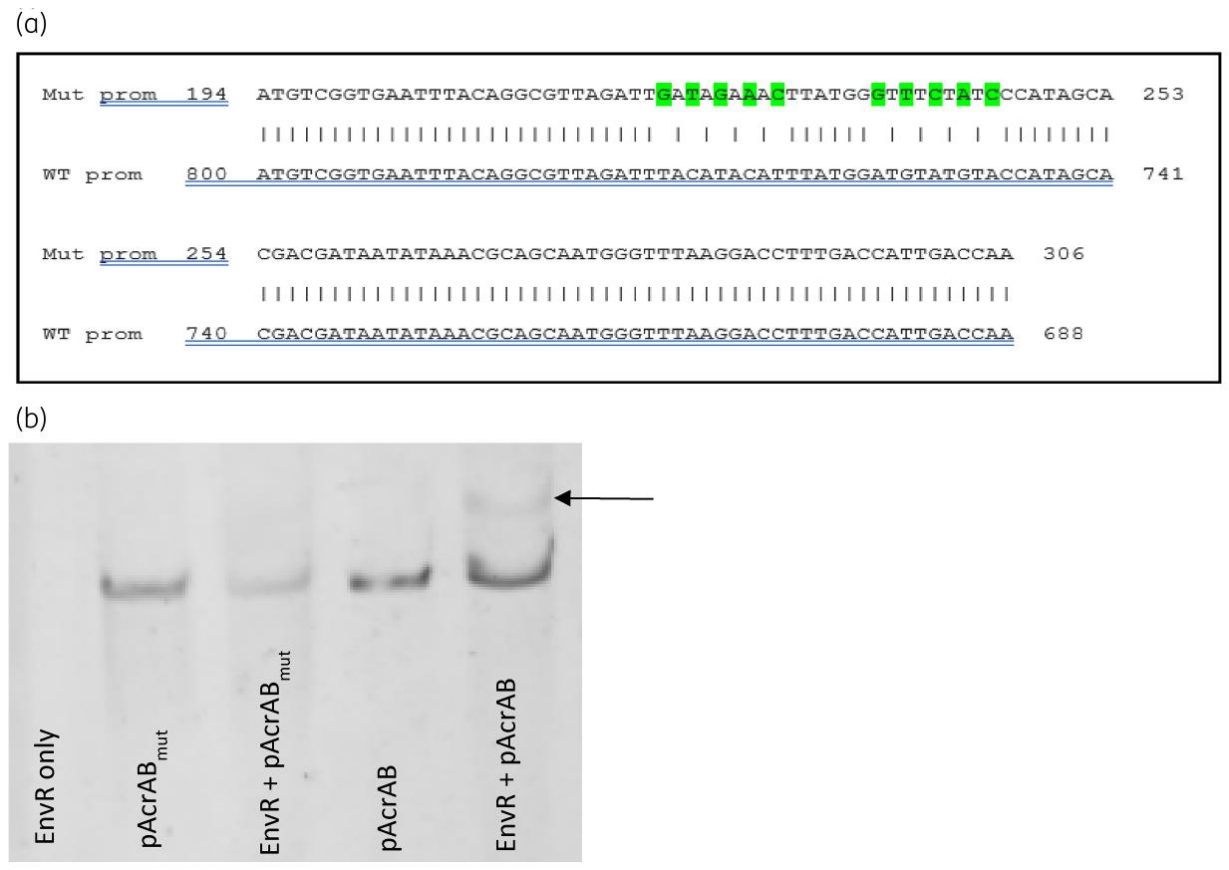
Confirmation of the binding site of *Salmonella* EnvR. (a) The promoter of *acrAB* was synthesized containing the following mutations in the known palindromic binding site of AcrR: T29G, C31T, T33G, C35A, T37C, A44G, G46T, A48C, G50A, A52C. (b) The binding of EnvR to the WT or mutated promoter (*acrAB_mut_*) of *acrAB* as determined by EMSA. The binding of EnvR to the promoter of *acrAB* was abolished when these mutations were present. This figure appears in colour in the online version of *JAC* and in black and white in the print version of *JAC*.

Based upon the known consensus binding sequence of AcrR^29^ and the binding site for EnvR in *E. coli*,^15^ a 24 bp palindrome was detected within the promoter region of *acrAB* in *Salmonella* and this was predicted to be the binding site of EnvR. To investigate this, the corresponding residues on each side of the palindrome were mutated by site-directed mutagenesis [Figure 2(a)] and the mutated promoter was used in an EMSA to see whether this reduced binding of EnvR. *Salmonella* EnvR bound to the original promoter sequence, but mutation of the predicted palindrome prevented all binding [Figure 2(b)].

**Figure 3.**
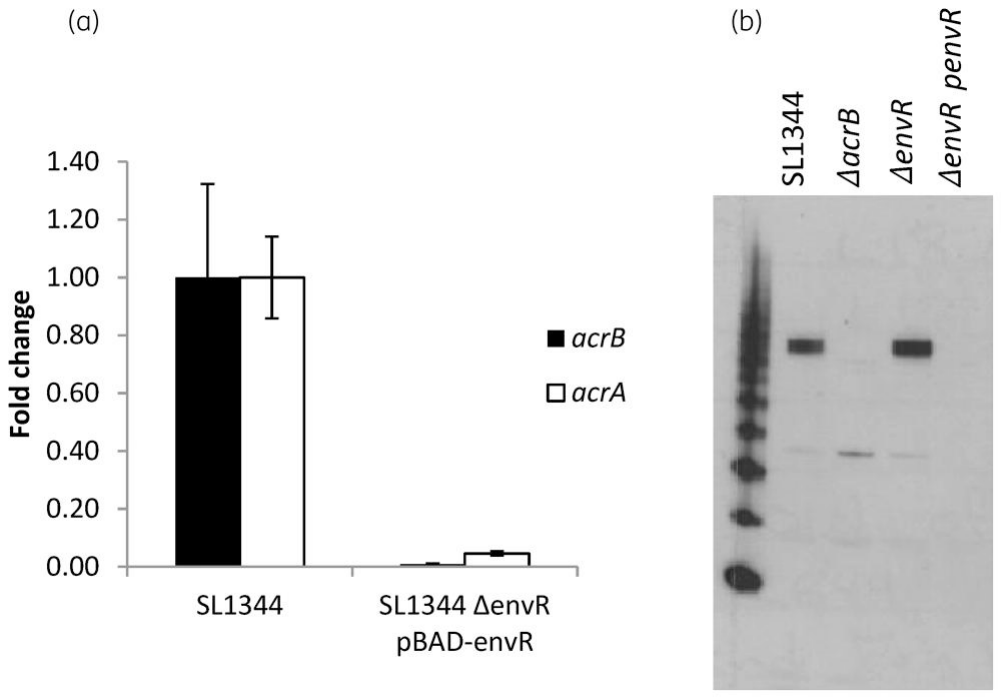
Overexpression of EnvR reduces transcription of *acrAB* and AcrB protein level. (a) Expression of *acrA* and *acrB* measured by RT–PCR in SL1344 and a strain overexpressing *envR*. Data shown are the mean of three independent biological replicates ± SE. (b) Western blot for AcrB showing decreased protein level when *envR* is overexpressed.

To determine whether the *in vitro* binding of AcrR and EnvR to the *acrAB* promoter translates into transcriptional regulation of *acrAB*, the *envR* gene from *S.* Typhimurium was overexpressed in SL1344 by cloning into an expression vector. Overexpression of *envR* reduced transcription of *acrAB* to 0.05-fold of that in SL1344 [Figure 3(a)]. In addition, western blotting with anti-AcrB antibody showed that *envR* overexpression also led to less AcrB protein [Figure 3(b)].

Together this shows that EnvR in *Salmonella* binds to the promoter region of *acrAB*, and that it can reduce transcription of *acrAB* and prevent production of AcrB protein.

### Overexpression of envR confers multidrug susceptibility, reduced motility and reduced ability to infect human cells in vitro

**Figure 4.**
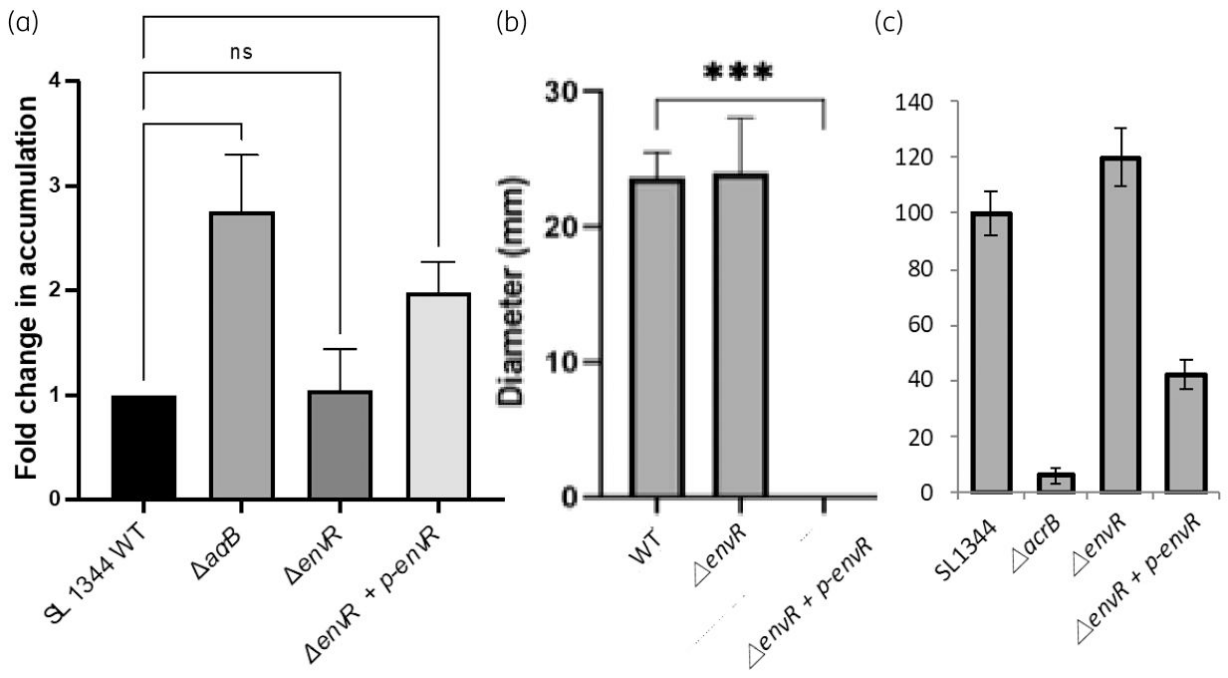
Phenotypic effect of inactivation or overexpression of *envR* in Salmonella SL1344. (a) Accumulation of Hoechst 33342 following inactivation or overexpression of *envR*. Data shown are the mean of three independent biological replicates ± SEM. (b) Swimming motility. Data shown are the mean ± SEM of three independent biological repeats. (c) Invasion of *Salmonella* strains into human intestinal cells (INT-407). Data shown are the mean ± SEM of three independent biological repeats. ns, not significant; ***P < 0.001.

Given that overexpression of *envR* reduces expression of AcrB, the effect on efflux-mediated phenotypes was measured. As previously described, deletion of *acrB* increased susceptibility to all AcrB substrates tested (Table 2).^22^ Deletion of *acrR* or *envR* or both *acrR* and *envR* (*ΔacrRΔenvR)* did not significantly alter antimicrobial susceptibility. This is likely to be because expression of *envR* in SL1344 in laboratory conditions is negligible so deletion has minimal impact. However, increased expression of either of the TetR regulator genes, *acrR* or *envR*, increased susceptibility of *Salmonella* to a range of AcrB substrates. Notably, increased expression of *envR* had a greater effect than increased expression of *acrR* for methylene blue, acriflavine, SDS and erythromycin, with MIC values for the *envR* overexpressing strain being <3-fold lower. Interestingly, this effect was substrate dependent. Accumulation of the fluorescent dye Hoechst 33342 is used as a measure of the level of efflux in bacterial cells.^25,30^ Increased expression of *envR* increased accumulation of Hoechst [Figure 4(a)]. Efflux level has also been associated with bacterial motility and the ability of Gram-negative pathogens to cause infection.^22^ Increased expression of *envR* completely prevented swimming motility [Figure 4(b)] and reduced invasion into INT-407 cells *in vitro* [Figure 4(c)].

**Table 2.** Impact of deletion or overexpression of efflux regulators on antimicrobial susceptibility.

	MIC (mg/L)									
	EtBr	CIP	NAL	CV	MB	SDS	ERY	CHL	TET	ACR
SL1344 (WT)	>1024	0.03	4	64	>1024	1024	64	4	1	256
△*acrB*	64	>0.008	2	4	32	256	4	2	0.5	32
△*acrR*	>1024	0.03	4	64	>1024	1024	64	4	1	128
△*envR*	>1024	0.03	4	64	>1024	1024	64	4	1	256
△*acrR*△*envR*	>1024	0.03	8	64	>1024	1024	64	4	0.5	128
△*acrR* pET20b*acrR*	**16**	>**0**.**008**	**1**	**16**	**256**	1024	64	**1**	0.5	**16**
△*envR* pET20b*envR*	**16**	>**0**.**008**	**2**	64	**16**	**256**	**16**	**1**	0.5	**4**

EtBr, ethidium bromide; CIP, ciprofloxacin; NAL, nalidixic acid; CV, crystal violet; MB, methylene blue; ERY, erythromycin; CHL, chloramphenicol; TET, tetracycline; ACR, acriflavine. Bold font indicates a ≥ 2 fold increase in MIC relative to parent strain without plasmid.

### EnvR inhibits RamA-mediated overexpression of acrB in a drug-susceptible and related MDR clinical isolate

**Figure 5.**
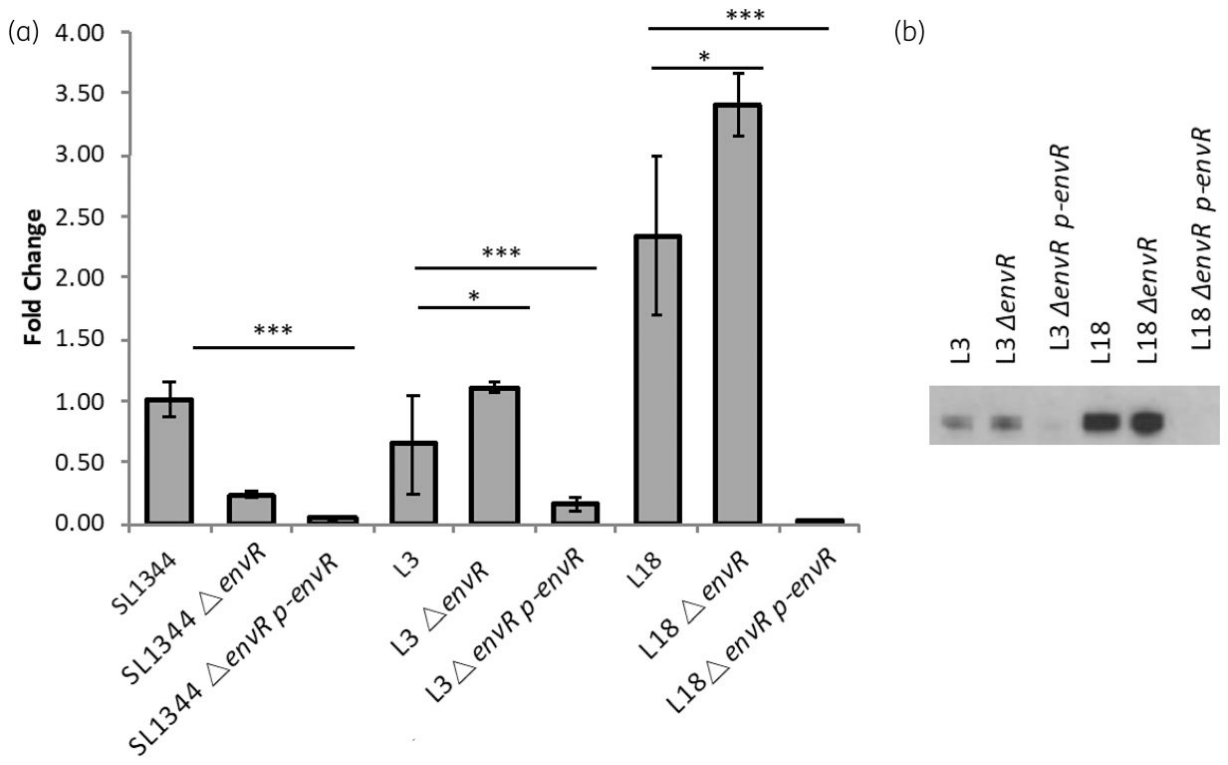
Effect of *envR* deletion or overexpression in different *Salmonella* backgrounds. (a) Fold change in gene expression *acrA* relative to SL1344 measured by real-time RT–PCR. (b) Western blot for AcrB. *P < 0.05; ***P < 0.001.

To confirm that overexpression of EnvR caused low *acrB* expression in the pre-therapy clinical isolate L3, *envR* was inactivated. Deletion of *envR* led to a 2.1-fold increase in *acrA* transcript in L3 *ΔenvR* compared with L3 [Figure 5(a)]. Western blotting for AcrB in the L3 *ΔenvR* strain showed a small increase in AcrB protein level [Figure 5(b)].

The *envR* gene was also deleted and overexpressed in L18. Inactivation of *envR* had little effect on the phenotype of L18. However, when *envR* was overexpressed there was no AcrB protein detectable by western blot. This shows that even with increased *acrB* expression (caused by overproduction of RamA) this can be completely inhibited by EnvR.

## Discussion

The clinical isolates L3 and L18 have been well studied previously and the mechanisms of MDR in the post-therapy isolate L18 are complex and multifactorial. We have previously reported the complete genome sequence of these clinical isolates^18^ and now report the complete transcriptome of this matched pair of clinical isolates from before and after a clinically validated course of antimicrobial treatment. The post-therapy isolate L18 was previously shown to have much higher expression of the efflux pump AcrB than the pre-therapy isolate L3.^19,20^ The regulation of *acrAB* expression has been recently reviewed in detail.^2,31,32^ Both the pre- and post-therapy isolates carry a mutation at codon 19 of *ramR*, introducing a stop codon and, therefore, both have higher expression of the major transcriptional activator RamA than the well-characterized isolate SL1344.^19^ However, the expression level of *ramA* and other regulators involved in regulation of efflux was broadly similar in L3 and L18, suggesting that this alone could not account for the differential *acrB* expression between L3 and L18. While comparison of L3 and L18 was illuminating, comparisons of both transcriptomes with a common reference strain, SL1344, also provided extra information to understand the biology. For example, the efflux pump *acrEF* and the cognate regulator *envR* appear to be expressed at low levels in L18 when compared with the pre-therapy isolate L3. However, a comparison with a common laboratory strain, SL1344, revealed that this may reflect a particularly high level in the pre-therapy isolate L3 rather than a low level in L18, as the expression of these components was high in L3 compared with SL1344.

Previous work in *E. coli* has shown that EnvR is a TetR family transcriptional regulator of *acrAB*, which binds to the promoter region upstream of *acrAB* and has a greater binding affinity than the local repressor AcrR.^15^ Here we have shown that EnvR is a potent repressor of *acrAB* transcription in *Salmonella*. It is proposed that EnvR could act as a switch between *acrAB* and *acrEF* transcription because transcription is initiated concurrently with *acrEF* and represses transcription of *acrAB.*^15^ Our data support this switch hypothesis because in the pre-therapy isolate, L3 expression of *acrEF/envR* was high while *acrAB* expression was low, presumably because it was being repressed by EnvR.

TetR family transcription factors are often thought of as simple, single-target regulators that usually regulate locally encoded genes. However, like EnvR, they are increasingly being shown to regulate multiple targets that can be encoded separately on the genome.^33^

The gain-of-function experiments where *envR* was overexpressed gave clear results with transcription, protein level and phenotype giving a coherent picture similar to genetic inactivation of *acrB*. However, loss-of-function experiments, when these TetR regulator genes were deleted, were much less clear, giving little or in some cases no effect. In the case of *Salmonella* SL1344, it is likely that deletion of *envR* had little effect because, due to H-NS silencing, it is not expressed under normal laboratory conditions.^34^

In summary, we have shown that EnvR is a potent repressor of *acrAB* transcription in *Salmonella*. Importantly, we can now show that efflux regulation by EnvR is clinically relevant as it explains the relatively low AcrB expression in a clinical pre-therapy isolate L3 compared with the post-therapy isolate L18. When overexpressed, EnvR can prevent expression of the major RND pump AcrB in an MDR clinical isolate and we have now shown that this increases intracellular accumulation, decreases bacterial motility and attenuates the ability to cause infection. Finding novel tools to increase EnvR expression may form the basis of a new way to prevent or treat MDR infections.

## Supplementary Material

dkac364_Supplementary_Data

## Supplementary data

Tables S1 and S2 are available as Supplementary data at *JAC* online.
