# Supplementary material for: EnvR is a potent repressor of acrAB transcription in Salmonella
Source: J Antimicrob Chemother. 2022 Oct 29;78(1):133–40. doi: 10.1093/jac/dkac364 (PMC9780535; doi:10.1093/jac/dkac364)
Supplement: dkac364_Supplementary_Data [file dkac364_supplementary_data.docx]

Table S1.

| **Strain code** | **Genotype (+plasmid)** | **Resistance** | **Reference** |
| --- | --- | --- | --- |
| **SE01 / WT** | *S. enterica* serovar Typhimurium SL1344 | - | Wray and Sojka, 1978 |
| **SE02** | SL1344 *ΔacrB* | - | Eaves et al., 2004 |
| **SE24** | SL1344 *ΔacrR* | - | This study |
| **SE35** | SL1344 *ΔacrR* + pET20b *acrR* | Amp^R^ | This study |
| **EC143** | BL21 (DE3) + pLysS | Cam^R^ |  |
| **EC144** | BL21 (DE3) + pLysS + pTrc acrR (+6xhis tag) | Amp^R^  Cam^R^ | This study |
| **SE23** | SL1344 *ΔenvR* | - | This study |
| **SE34** | SL1344 *ΔenvR* + pET20b *envR* | Amp^R^ | This study |
|  | BL21 (DE3) pLysS + pTrc *envR* (+6xhis tag) | Amp^R^  Cam^R^ | This study |
| **SE57** | SL1344 *ΔacrR* *ΔenvR* | - | This study |

Table S2

| Primer name | Sequence |
| --- | --- |
| EnvR KO Fwd | CGGCGTTGCCAACACGACGTTGAATGATATCGCTGATGCCGTGTAGGCTGGAGCTGCTTC |
| EnvR KO Rev | CGCTAAAACTACCATGCAGAATAATTAATATGACATCCAGGGGAATTAGCCATGGTCCAT |
| Cloning *envR* into pET20b NdeI F | GGGGGGGGcatAtgGcgAagAaaAcgAagGcgGat |
| Cloning *envR* into pET20b HindIII R | GGGGaagctttcAggCttCttCcgCctGttGttCatTtgG |
| Cloning *acrR* into pET20b NdeI F | GGGGGGGGcatATGGCACGAAAAACCAAACAACAA |
| Cloning *acrR* into pET20b HindIII R | GGGaagcttTCAGGGGGAGCCGTTGACCGTCGA |
| Promoter of *acrAB* Fwd (for EMSA) | TCCCAGATCTCACTGAATA |
| Promoter of *acrAB* Rev (for EMSA) | TCAATGGTCAAAGGTCCT |
| *acrA* RT-PCR forward | GTAATTTCGTTGAGGGAAGTGA |
| *acrA* RT-PCR forward | TCGTAAGTCGCCTGGTAG |
